# Evaluating the Performance of a Semiaromatic/Aliphatic Polyamide Blend: The Case for Polyphthalamide (PPA) and Polyamide 4,10 (PA410)

**DOI:** 10.3390/polym13193391

**Published:** 2021-10-02

**Authors:** Mateo Gonzalez de Gortari, Feng Wu, Amar K. Mohanty, Manjusri Misra

**Affiliations:** 1 School of Engineering, Thornbrough Building, University of Guelph, Guelph, ON N1G 2W1, Canada; mgonza07@uoguelph.ca (M.G.d.G.); fengwu@uoguelph.ca (F.W.); mohanty@uoguelph.ca (A.K.M.); 2 Bioproducts Discovery and Development Centre, Department of Plant Agriculture, Crop Science Building, University of Guelph, Guelph, ON N1G 2W1, Canada

**Keywords:** polyphthalamides, polyamide 4,10, high-performance polymer

## Abstract

This paper studies the structure–property–processing relationship of polyphthalamide (PPA) PPA/polyamide 4,10 (PA410) blends, via co-relating their thermal-mechanical properties with their morphology, crystallization, and viscoelastic properties. When compared to neat PPA, the blends show improved processability with a lower processing temperature (20 °C lower than neat PPA) along with a higher modulus/strength and heat deflection temperature (HDT). The maximum tensile modulus is that of the 25PPA/75PA410 blend, ~3 GPa, 25% higher than neat PPA (~2.4 GPa). 25PPA/75PA410 also exhibits the highest HDT (136 °C) among all the blends, being 11% more than PPA (122 °C). The increase in the thermo-mechanical properties of the blends is explained by the partial miscibility between the two polymers. The blends improve the processing performance of PPA and broaden its applicability.

## 1. Introduction

In the current era, plastic is one of the most abundant materials produced by humanity. According to the European Association of Plastics Manufacturers (PlasticsEurope), 359 million tonnes of plastic were produced in 2018 [1]. So-called commodity plastics, such as polystyrene (PS), polyethylene (PE), polyvinyl chloride (PVC) and polypropylene (PP), all used in applications where mechanical characteristics or melting points do not have high requirements, accounted for 78% of the worldwide demand of plastics in 2018 [1]. However, more niche plastics exist, as they are engineered and used to fulfill specialized applications that commodity plastics cannot fulfill. These are commonly referred to as “engineering plastics” and include several of the currently available nylons that have increased mechanical or thermal properties, such as higher melting points. However, there are situations where the properties of engineering plastics are not enough, so instead, a subgroup of these, called “high-performance engineering polymers” are utilized. Though no formal definition exists for this group of polymers, Ullman’s Encyclopedia defines them as polymers that are able to maintain acceptable mechanical characteristics at temperatures above 150 °C [2].

It is within this category that polyphthalamide (PPA), a thermoplastic polymer, is located. Parts produced from this polymer are used to replace metals in applications that require high temperature resistance, such as oil coolers and housings laptops, for PPA grades that are flame resistant [3]. Unlike many other polymers, PPA is an umbrella term for a family of plastics. According to ASTM International, a polymer must have at least 55% molar mix of terephthalic and/or isophthalic acids [4] for a manufacturer to refer to a polymer as PPA. Among their general characteristics, PPAs maintain their mechanical properties at high temperatures and are resistant to a wide variety of chemical attacks [3]. Due to their high melting point (it can range from 285 to 315 °C, depending on the grade and manufacturer’s composition [3]), they also require a higher temperature to be processed and molded, compared to traditional commodity plastics. As such, additional energy use and expenses are incurred. If PPA could be modified to increase its flowability and lower its melting temperature, it could expand the horizon of possible applications and reduce the energy required during its processing.

As in many other areas of material science and engineering, the first instinct to improve or modify the properties of a polymer such as PPA would be to mix it with a second polymer that has complimentary properties. Preferably, this would be with one that has a lower melting point, to obtain a blend that has the advantages of both components, with none of the weaknesses.

Plastics wholly produced from renewable sources are found to have a lower environmental impact [5], such as the biobased polyethylene terephthalate (PET) [6]. Many other examples of the incorporation of natural feedstocks exist, including PPAs that incorporate bio-derived raw materials [7,8]. Currently, however, most of the available supply of PPA is based on oil-based or non-renewable, unsustainable sources. As such, increasing the amount of biobased material incorporated into the PPA matrix, while not decreasing any of the mechanical properties, or even increasing them, would result in a product that would be more acceptable to both consumers and producers. 

Looking at the available bio-based polymers, as well as those derived from sustainable sources, PA410, was selected and blended with PPA. Developed by the DSM company, this polyamide is derived from 1,4-diaminobutane and sebacic acid. PA410 exhibits high stiffness (modulus of 3 GPa), acceptable elongation at break (30%), high heat deflection temperature (160 °C) and, compared to PPA, a lower melting point (250 °C). It has been previously blended with polyamide 6,10 [9], compounded with micro fibrillated cellulose [10], and multi-walled carbon nanotubes [11]. Doing so aims to research the necessary conditions to incorporate the cellulose and to improve both the mechanical and electrical conductivity properties, respectively. 

Most biopolymers are currently being investigated in order to replace commodity plastics in single-use applications or in biomedical applications. The relative high cost of polymers and the expected low cost of the intended applications make this substituon a challenge. If bio-based polymers cannot currently compete in low-cost applications, they could have success in supporting or substituing more niche and speciallized applications, in higher-cost but necessary applications. It is expected that the blending of PPA with PA410 will improve the processability of PPA without sacrificing the thermal-mechanical advantages that PPA has, as well as expanding the possible applications of PA410, such as for automotive purposes, under the hood parts. As the neat PA410 has some mechanical properties above that of PPA, it is also desirable to obtain blends that will be above that of neat PPA To our knowledge, no previous research on a PPA/PA410 blend has been disseminated in a peer-reviewed scientific journal.

## 2. Materials and Methods

PPA (Dupont company, Midland, Michigan, USA, grade Zytel HTFNFE8200 NC010) and PA410 (DSM company, Heerlen, Netherlands, Limburg, grade Ecopaxx Q210E-H) were dried for four hours, at 120 °C and 80 °C, in order to keep the moisture content under the manufacturer’s recommended limit (0.15% and 0.1%, respectively). Using the wt% indicated of PPA and PA410 in Table 1, the appropriate amounts of each polymer were weighted and hand mixed, before being manually injected into a micro-compounder from the DSM company (Xplore Instruments, Sittard, Limburg, The Netherlands), which has a chamber with a 15 cm^3^ capacity and a twin-screw configuration. After two minutes of residence, with the speed of the screws at 100 rpm and with the temperature set on the melt compounder, as indicated by Table 1, the polymer melts were injected into heated molds at 100 °C, via the use of a cylinder-shaped injector (Xplore Instruments). The pressure used was 16 bar, for a total of 30 s of holding time. ASTM standard samples for impact energy, tensile and flexural mechanical tests were produced.

### 2.1. Mechanical Analysis

Type IV, ASTM D638-14, tensile samples were tested in an Instron Universal Testing Machine 3382 (Norwood, Massachusetts, USA, Instron company) equipped with a video extender using a total of five specimens for each blend. To ensure that the samples broke in a period between 30 s to 5 min (as indicated by ASTM D638-14), the crosshead speeds of blends were controlled at 5 mm/min while the speeds for neat PPA and PA410 were 50 mm/min. The results presented are the average of five tested samples.

Using a crosshead speed of 14 mm/min, the flexural properties of the blends were tested under ASTM D790-15 procedure B, tested in an Instron Universal Testing Machine 3382 as well. This speed is the result of the calculation of using samples with standard dimensions. The results as shown were the product of averaging five total samples.

Impact energy was tested under an ASTM D256, using a Zwick/Roell (Ulm, Baden-Württemberg, Germany) Impact tester. Notching the plastic pieces as per the standard, five specimens were tested with a 2.75 J hammer, with the individual results being averaged.

### 2.2. Thermogravimetric Analysis (TGA)

Cut-off samples from the mold-injected samples (ASTM D790-15), weighing between 15 and 20 mg were placed on a platinum plan and heated, starting the process at room temperature, using a heating rate of 10 °C/min, and reaching a final temperature of 700 °C, with the use of a flow of 50 mL/min of nitrogen gas. The apparatus used was a TGA Q500 (New Castle, Delaware, USA, TA Instruments company).

### 2.3. Differential Scanning Calorimetry (DSC)

Samples between 5 and 10 mg were cut and placed inside a sealed aluminum pan, then placed inside a DSC Q200 (TA Instruments). The analysis was conducted using a heating, cooling, and heating program, with the following parameters: 0 °C was established as an initial temperature, the temperature held constant for two minutes. After, heating the sample with a heat rate of 10 °C/min, the samples reached 350 °C, which was maintained for two minutes. The cooling cycle was then established by cooling the samples at a rate of 10 °C/min to a temperature of 0 °C, and once again, after two minutes of maintaining the temperature constant, a final cycle of heating, once again to 350 °C, at a 10 °C/min heating rate.

### 2.4. Scanning Electron Microscopy (SEM)

Impact samples previously broken from all blends, were gold coated under an Argon atmosphere for 12 s and were observed using a Phenom ProX microscope from Phenom-World BV (Eindhoven, North Brabant, Netherlands). A voltage of 15 kV was used, and magnifications of 500×, 5000× and 15,000× were used in order to study the blends morphology.

### 2.5. Atomic Force Microscopy (AFM)

A microtome (Wetzlar, Hesse, Germany, Leica company) was used to ensure that each blend would have a smooth surface, at room temperature. The surface was probed using a Multimode-8 AFM instrument (Bruker Nano Inc. Company, Billerica, MA, USA) using force taping mode, at a scan rate of 0.401 Hz.

### 2.6. Dynamic Mechanical Analysis (DMA)

Using untested bars of the same dimensions, as the ones obtained for the impact energy analysis, samples were placed inside a DMA Q800 (TA Instruments). For the measurement of HDT, a force was imparted upon the bars to cause a stress of 0.455 MPa, and they were heated up to 30 °C below their melting points, as observed under DSC or until the strain measured exceeded 0.22%. The HDT was taken at 0.2% strain. For their viscoelastic properties, an initial temperature of −30 °C was established. A thermal program was then followed, heating the materials at 3 °C/min, for a final temperature of 220 °C. An oscillating frequency sweep of 1 Hz, with an amplitude of 15 μm was employed.

### 2.7. Polar Optical Microscopy (POM)

Broken impacted samples were cut down with a metal cutter and placed in a glass sample holder. They were then melted with a second plate of glass placed over it until a thin layer of the blend formed. The samples were then observed under a polarized optical microscope (Nikon Instruments, Tokyo, Kantō, Japan), heating them to 310 °C at a 50 °C/min rate, then cooled down to their glass transition temperature (*T_g_*, as calculated by DMA) at 30 °C/min, the temperature then set constant for 3 min, finally cooling them down to room temperature, using a 50 °C/min rate. The images were taken at 20× magnification at room temperature.

### 2.8. Rheology

Samples of each blend were cut from injection molded specimens (having the same dimensions as those used in in ASTM D790-15), then placed at a parallel plate rheometer, MCR302 (Graz, Styria, Austria, Anton Paar company). Frequency sweeps were performed from 600 to 0.1 rad/s, at 310 °C, 1% strain for all blends under nitrogen protection.

### 2.9. Fourier-Transform Infrared Spectroscopy (FTIR)

A sample of each blend and neat polymer was analyzed trhough FTIR, using the attenuated total reflection infrared spectra, with a Nicolet iS20 spectroscope (Thermo Scientific, Waltham, MA, USA). 128 scans were employed and averaged. Each scan was taken from a wavenumber of 4000 cm^−1^ to a wavenumber of 525 cm^−1^, with a 0.5 cm^−1^ resolution for each scan.

## 3. Results and Discussion

### 3.1. Mechanical Properties

**Figure 1:**
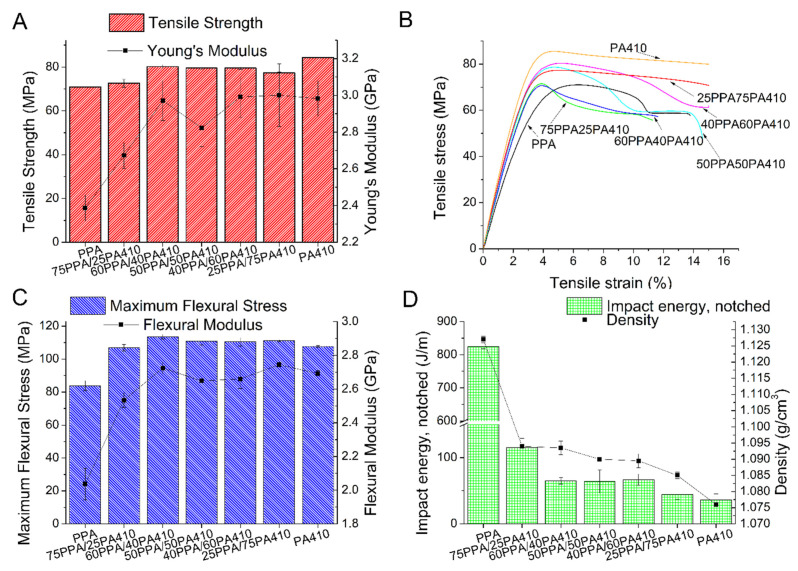
Mechanical properties of polymer blends of PPA/PA410. (**A**) Tensile properties; (**B**) tensile stress-strain curve; (**C**) flexural properties; and (**D**) impact energy and density.

An increase in both the tensile strength and Young’s modulus of the samples was observed, as the content of PA410 increased with respect to PPA, as shown in Figure 1A. The respective tensile stress–strain curve is shown in Figure 1B. Flexural properties presented in Figure 1B show a similar trend, as a result of the higher modulus/strength of PA410 compared to that of PPA. However, with the addition of PA410, the impact strength of the blends is reduced because of the lower toughness of PA410, as seen in Figure 1D. The density experiences a dramatic drop with any of the measured additions of PA410, as shown in Figure 1D. Similar results were found with a PA6I/PA6T polymer matrix (ratio of 1:2) and a polyamide 6 (PA6), in which the blends of the two compatible polymers had higher mechanical properties compared to the neat polymer with the lowest mechanical properties [12], which in their case was with PA6. Other researchers found the case to be the same, when using cast-films, again with PA6 and PA6T/PA6I [13]. The researchers attributed this increase in the mechanical properties as the stiffening of the molecular chains, though in their case, it was the PA6I/PA6T polymer chains causing this effect on the PA6. Finally, the elongation at break went from 19.64% ± 3.28% with neat PPA, to 10.55% ± 2.04% with 25% of PA 410, 5.66% ± 1.35% with 40% of PA410, 15.53 ± 3.55 with 50% of PA410, 17.05 ± 4.25 with 60% of PA410, 19.87 ± 3.74 with 75% PA410 to 36.71 ± 4.62 with neat PA410. The sudden decrease, in particular for 60PPA40PA410, suggest that there is incompatibility and bad adherence between the two polymers. The results presented in this study suggest that the PA410 stiffened the PPA. Though not commercially disclosed, the type of PPA used in this study is composed of a 50:50 mix of 2-methylpentamethylene diamine and hexamethylene diamine [3], while all the monomers of the PA410 are linear. In the case of these blends then, the increase in linear monomers increases the possibility of chain packing. Additionally, as was reported elsewhere, transamidation of PPA with other polyamides was observed [14,15], in which block copolymers are formed. As the length of the aliphatic chains decreases between the aromatic rings, this could also help explain this increase in mechanical properties. However, we can see that for the 50PPA/50PA410, there is marked decrease in its mechanical properties, compared to the immediate blends. This suggests that there is some inherent incompatibility between the polymers, at least when the ratio between them is 1:1. This possible limit to the miscibility between them will be further explored and discussed in subsequent sections of this paper.

The simplest way to predict this type of results is by using the rule of mixtures. The upper bound (the maximum), as defined by the rule of mixtures, can be stated as Equation (1) [16]:

(1) $$P=\sum^{\;}f_{i}P_{i}$$

In which P is the property of the blend being analyzed, $f_{i}$ is the fraction of the *i*^th^ component and $P_{i}$ is the property of the neat *i*^th^ component.

**Figure 2:**
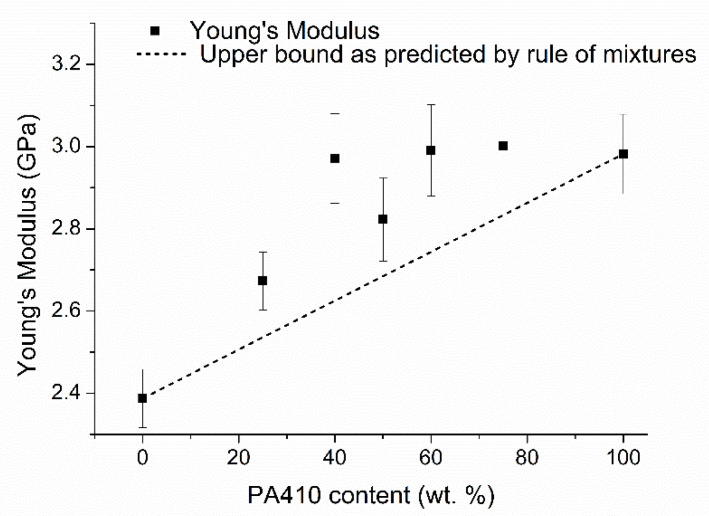
Comparison between the theoretical upper bound of the Young’s Modulus predicted by the rule of mixtures and the results of the measured blends, as a function of wt% of PA410.

As seen in Figure 2, the Young’s modulus of the blends is above what would be predicted by the rule of mixtures, meaning that the polymers are interacting with each other in some manner. This same behavior can be extended for the flexural properties as well. Previously published studies suggest that the good compatibility between a PPA and other polyamides can be explained through a transamidation process [14,15], in which the PPA and the polyamide react and switch monomers between the polymer chains, resulting in copolymers. However, the short amount of residence time employed to produce the blends in this study means that the percentage of randomness and degree of transamidation in the studied blends would be low [17]. As for PPA, its solubility does not depend on the presence or location of the phenylene group within the polymer chain, but on the conformation of the rest of the polymer chain [18]. Finally, hydrogen bonds are also known to occur between polymer chains that contain amide bonds, so PPA and PA410 could be interacting through this mechanism.

### 3.2. Thermal Analysis

**Figure 3:**
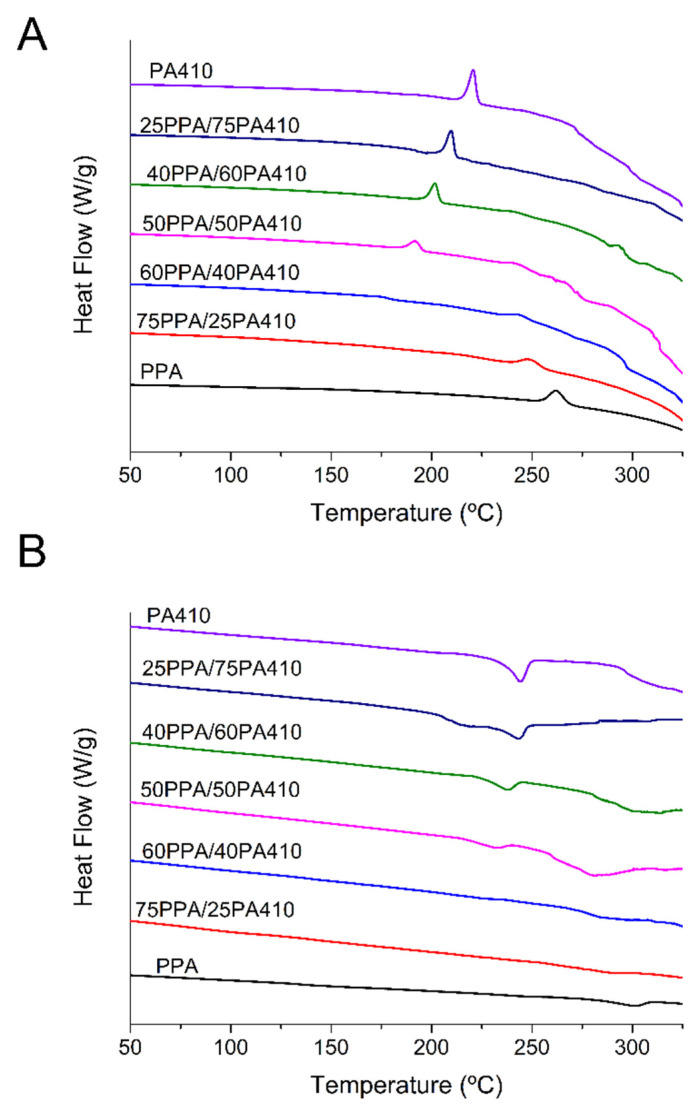
DSC curves of the PPA, PA410 and their blends: (**A**) first cooling cycle; (**B**) second heating cycle. Both cycles were measured at a rate of at 10 °C/min. Exotherms are up.

Figure 3 shows the thermal behavior of the blends. The first cooling curve (Figure 3A) shows a decrease in the crystallization temperature to a minimum for the 50PPA/50PA410 blend, after which, as the content of PA410 increases, so does the crystallization temperature. A decrease in the melting temperature of polymer blends, compared to the blend with the highest melting point is typical of miscible blends, especially when an amorphous polymer is added to a semicrystalline one, as the result of kinetic, morphological and thermodynamic factors [19]. The depression of the melting point was observed in other PPA blends with polyamide 6,6 and polyamide 6,10 [15]. In Figure 3B, the addition of low contents of PPA to PA410 and vice versa, shows a small depression in melting points, while the blends with a higher content of either show double melting points. According to the literature, this can indicate either a separate crystallization of each polymer, but it can also be due to the re-crystallization of thinner imperfect crystals, into thicker, more perfect ones. However, the distance between the two melting points is large, which would point out to immiscibility in the blends closer to a 50:50 ratio between PPA and PA410. The addition of PA410 also shows a reduction in the crystallization temperature. A possible explanation is that within certain ranges of the content of each polymer, each one acts as a polymer matrix, with the remaining polymer being dissolved in it. Eventually, a point is reached in which they both hinder the crystallinity/crystal growth of the opposite polymer, or they are both too abundant to form a particular phase or structure within the surrounding matrix. A similar case with PA46 and PA6I (poly hexamethylene isophthalamide) was reported by Eersels et al. [20], the authors found that the content of the amorphous PA6I did not significantly affect the crystal growth of the linear PA46, until a certain threshold content of above 70% wt. PA6I was introduced in the blend. 

In order to investigate their thermal behavior, Equation (2), was used to calculate the percentage crystallinity (*χ_c_*):

(2) $$\chi_{c}=\frac{\Delta H_{m}-\Delta H_{c}}{W_{f}*\Delta H_{m}^{0}}*100$$

Where $\Delta H_{m}$ is the melting enthalpy, $\Delta H_{c}$ is the crystallization enthalpy, $W_{f}$ is the weight fraction of the polymer being considered, and $\Delta H_{m}^{0}$ is the melting enthalpy of a theoretical 100% crystalline polymer. A reference or experiment to calculate the $\Delta H_{m}^{0}$ for neat PPA has not been found, and previously published works have also not been able to find a source [21]. However, it has been calculated for neat PA410 (269 J/g [9]). Therefore, only the crystallinity of PA410 was calculated here, using the single melting peak for all the apparently miscible blends and the lower temperature one for the ones with double melting peaks, with the results shown in Table 2. The results show that the crystallinity of the PA410 increases when it is at lower concentration in a PPA matrix, that is, the restriction of PPA on of the PA410 may be inducing a more crystalline structure than the polymer by itself. Further additions decrease it until it reaches a minimum at 50PPA/50PA410, to then increase at 40PPA/60PA410, to then decrease and be relatively constant as the wt% of the PA410 again reaches the neat polymer. The linear polyphthalamide chains form crystalline structures that cannot be melted, as their melting temperature is too close to their degradation temperature. This was previously explored by Shashoua and Eareckson, whose research found that spin fiber techniques were necessary to synthetize polyphthalamide with short methylene chains [22], while only degraded polymer chains could be obtained if condensation polymerization techniques were employed, as pointed out by Edgar and Hill [23]. This could then induce an increase in the crystallinity of the PA410 region, with the more linear parts of the PPA chain, while the rest of the branched comonomers are rejected, forming a possible co-crystallization. Further, the addition of the PPA to PA410 can limit the solidification rate due to restriction in chain mobility, which is seen by the downward shift of the crystallization temperature of the PA410 crystals.

**Figure 4:**
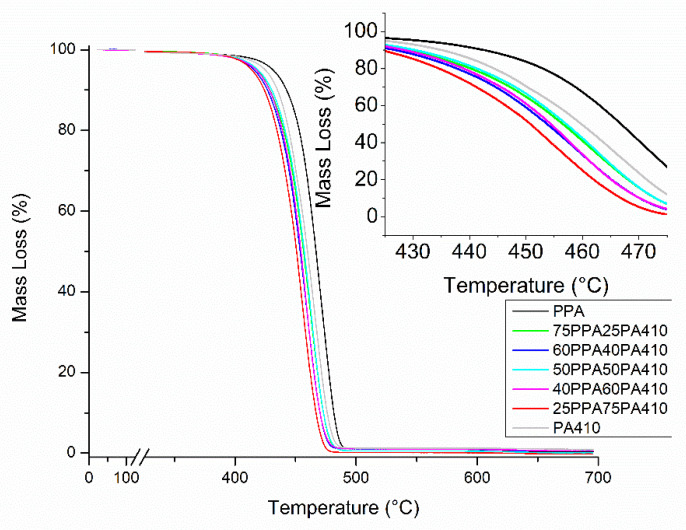
TGA curves of neat polymers and the prepared blends.

Both neat polymers, PPA and PA410, show a higher thermal stability compared to all the blends, in all the temperatures at which they lose 2%, 5% and 10% of their weight, as well as their maximum degradation temperature, as shown in Table 3. The TGA also shows us that although some discoloring might happen to the PA410 (as was previously reported [9]), at being melted and mixed at such a high temperature compared to its melting point, the PA410 is not suffering a significant amount of thermal degradation and can be safely processed at such temperatures. The TGA curves of individual samples of the neat polymers and the blends can be seen in Figure 4. The literature indicates that the complex chemical reactions that take place during the thermal decomposition of a polymer blend, are affected by a variety of factors, such as the molecular weight of the polymer, its chemical structure, its degree of crystallinity, etc. This means that thermal stability cannot be used as evidence of the miscibility, immiscibility or partial miscibility between two polymers, as blends can have a synergistic, antagonistic or additive thermal behavior when blended [24,25]. The *T_g_* of the blends could not be detected through DSC.

The HDT of the blends increased as a function of the amount of PA410, once the 50/50 threshold was passed, with the first initial blend having a slightly lower HDT than that of neat PPA, though due to the high margin of error in both measurements, the change is not significant. This means that the HDT of the blend appears to be a function of PA410 being the matrix, possibly due to its close molecular packing, because of the linear monomers in its structure. As seen in a study in which two different PPAs were blended with PA6 and PA66 [26], the HDT of the blends either remained the same or was lower than that of the PPA, for blends in which the wt% of the aliphatic polyamides was less than 50%. This shows that the PPA is not significantly affected by the addition of PA410, at least in terms of HDT, and it is only when the polymer matrix changes the PA410, that a change is observed.

### 3.3. Morphology Characterization

SEM microscopy is used in polymer science and engineering to look at the morphology of blends. When PPA was melt-mixed with polybutylene succinate (PBS) as the polymer matrix, globules of the PPA were visible [27]. A similar case can be seen with aluminum diethyl phosphinate (AlPi) and PPA, showing the embedded particles [28]. All the micrographs, shown in Figure S1, are not distinguishable from each other. All the produced blends showed a similar homogenous structure, with no clear distinctions between the two different polymers. No globules, streaks or regions could be easily discerned, nor any phase separation; the only features that can be seen are the striations caused by the impact test and the microvoids from the fractures in the polymer matrix. A similar result was found in a study in which PPA was melt-mixed with polyamide 6 [29], the morphology of the samples showed the blends all having a homogenous structure, with no apparent phase separation between the two polymers.

Using POM, the internal structure of the blends and neat polymers can be seen in Figure S2. No crystalline regions within the size of the microscope were detected. While a change in the size and nature of the structures can be seen, in which the addition of PA410 changes the apparent small spherulites (as seen in other cases of POM of the neat PPA [30]) into a continuous phase, no particular phase separation nor structure are obviously seen. To capture smaller microstructure differences, AFM images were taken. 

**Figure 5:**
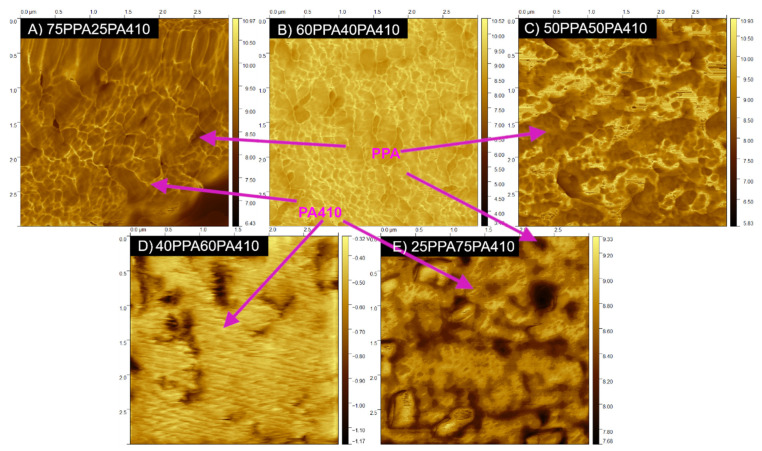
AFM modulus images of all the PPA/PA410 blends: (**A**) 75PPA25PA410; (**B**) 60PPA75PA410; (**C**) 50PPA50PA410; (**D**) 40PPA60PA410; (**E**) 25PPA75PA410. The dimensions of all squares are 3 µm × 3 µm.

Figure 5 shows the phase dispersion for the blends with 25 to 50 PA wt% PA410. As PA410 has a higher modulus, it is reflected as a yellow color in the AFM modulus images, evenly dispersed throughout the whole polymer matrix. As the content of PA410 increased beyond 50 wt% and becomes the main polymer matrix, a phase inversion occurred in the blends with 60 and 75 wt% of PA410, the PPA becoming a continuous phase dispersed in the PA410 polymer matrix. Several studies of miscible PPA and PA blends show similar results [13,15], an increase in the roughness of the surface of the films and a fibrillar morphology was observed. The lamellae that could be observed in one of the studies were in the order of 7 nm [13].

### 3.4. Viscoelastic Characterization

**Figure 6:**
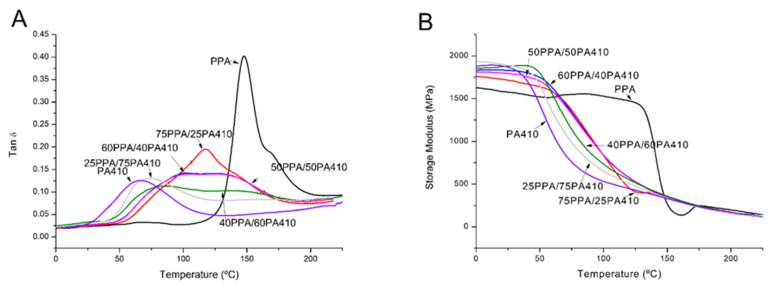
DMA curves of the PPA, PA410 and their blends; (**A**) dependence of tan (δ) on the temperature; (**B**) dependence of storage modulus on temperature.

The viscoelastic properties of the neat polymers and the prepared blends can be seen in Figure 6. The neat PPA shows a significant shift on the storage modulus behavior from the rest of the blends and the neat PA410, as seen in Figure 6B. Specifically, its storage modulus starts lower than the rest, at −20 °C, but it only starts decreasing at 125 °C, unlike the other blends whose storage modulus decreases around 50 °C. As the storage modulus corresponds to the elastic behavior of the polymer, it indicates that PPA remains a tougher polymer at higher temperatures, even though the rest of the materials have a higher initial storage modulus. The *T_g_* of all the blends was taken from the maximum of the peak of the Tan δ signal and is shown in Table 4. It appears to depend on the content of PPA, because as the wt% of PPA decreases, so does the *T_g_* of the blend. Similar results in the reduction of the glass transition temperatures were found in the case of blends of PPA with PA6 and PA6,6, with similar reductions in the plateau section, as seen in the behavior of the storage modules over a range of temperatures [26]. The author stated this as evidence of co-crystallization between their chosen PPA and the aliphatic polyamides. 

However, as it can be clearly seen in Figure 6A, not all the polymer blends show a distinct single peak nor two distinct ones in between the two neat polymers, but rather a flattened peak; a signal with a plateau. Two samples for each blend were measured for the DMA analysis, both showing the same behavior, so sampling error cannot explain this phenomenon. As such an explanation must be offered. According to Kaplan [31], this is evidence that the miscibility of the PPA with PA410 is not complete for all the ratios, but rather that it can be divided in two: (a) from neat PA410 up to 25PPA/75PA410, and from 75PPA/25PA410 to neat PPA, alongside the SEM we can say that the two polymers are completely compatible, as the tan δ peaks shown are sharp; (b) from 40PPA/60PPA410 to 60PA/40PA410, the two polymers are semi compatible, as seen from the broad, nondistinctive peaks in tan δ. As previously mentioned, the increase in mechanical properties, despite their immiscibility, could be explained as hydrogen bonding between the two polymers.

### 3.5. Rheological Properties

**Figure 7:**
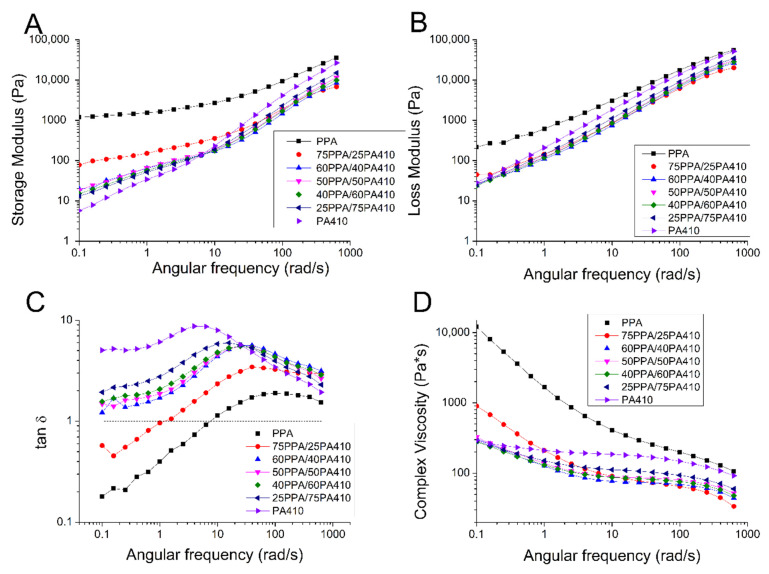
Dependence of rheological properties of the PPA, PA410 and their blends on angular frequency: (**A**) storage modulus; (**B**) loss modulus; (**C**) tan delta; and (**D**) complex viscosity.

The rheological behavior of the neat polymers and blends can be seen in Figure 7, via plotting complex viscosity, loss modulus and storage modulus against the angular frequency. PPA shows significant shear thinning, as its complex viscosity decreases two orders of magnitude from low frequency to high frequency, as shown in Figure 7D. With increasing PA410, the viscosity of PPA decreases because of the low viscosity of PA410 itself. At the same time, with the increase of PA410, the shear thinning of the blends becomes less obvious, particularly in the low frequency regions. The different shear thinning behavior is normally a result from changes in the conformation of polymer chains in the system. The introduction of PA410 chains, which is non-sensitive to the shear rate, disrupts the molecular chain entanglement or conformation of PPA. The chains require less shear to be disentangled, which explains the change of the blends to a Newtonian-like fluid. It is interesting to find that the viscosities of the blends at a high shear rate are lower than those of neat polymers, i.e., PPA and PA410 themselves. Therefore, the viscosity of PPA was dramatically decreased, even with only 25 wt% of PA410.

**Figure 8:**
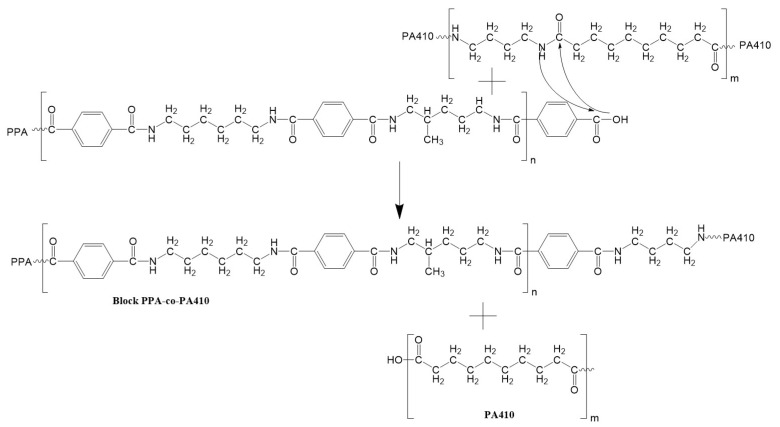
Schematic showing the formation of a block copolymer, between PPA and PA410.

Figure 7A,B, both show the decrease in the loss and storage modulus of the PPA with added PA410, probably due to the lower storage/loss modulus of the neat PA410. According to Zytner et al. [32], the tan δ is more sensitive than either loss or storage modulus and can help to understand the viscoelastic properties of polymer blends. Therefore, the ratio of loss modulus/storage modulus, called tan δ, is shown in Figure 7C. As all blends have a tan δ above 1, all blends show predominantly viscous properties. This also means that no chain branching or cross polymerization occurred between PPA and PA410. However, Wu et al. [33] indicates that the presence of a peak is evidence of a network structure. The explanation of a peak is that PPA and PA410 could be exchanging monomers and becoming miscible within a certain ratio, as predicted by the transamidation theory. And the co-crystallization happening between PPA and PA410 is allowing the formation of a network structure, without chain branching or cross polymerization. A schematic showing the possible reaction and formation of a block copolymer between PPA and PA410 is shown in Figure 8. The formation of the equivalent copolymer is not shown.

**Figure 9:**
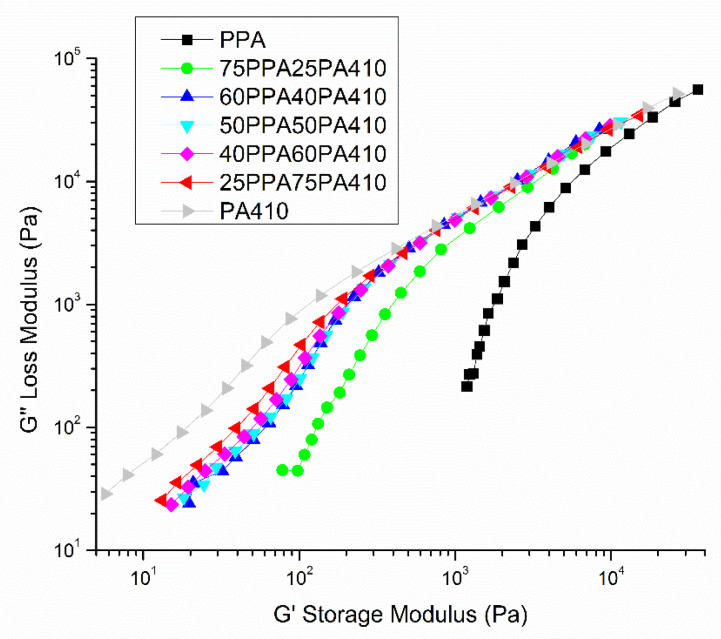
Cole–Cole plot of neat polymers and prepared blends.

After plotting a Cole–Cole curve, shown in Figure 9, all blends appear to have a semi-circle shape which would suggest that the blends are homogenous, as no shoulders are present within the data [34]. This means that although the polymers may not be completely miscible between each other, they were thoroughly dispersed between each other, providing further evidence of a network structure. However, further experiments would be necessary in order to refute or confirm these explanations.

### 3.6. FTIR Analysis

**Figure 10:**
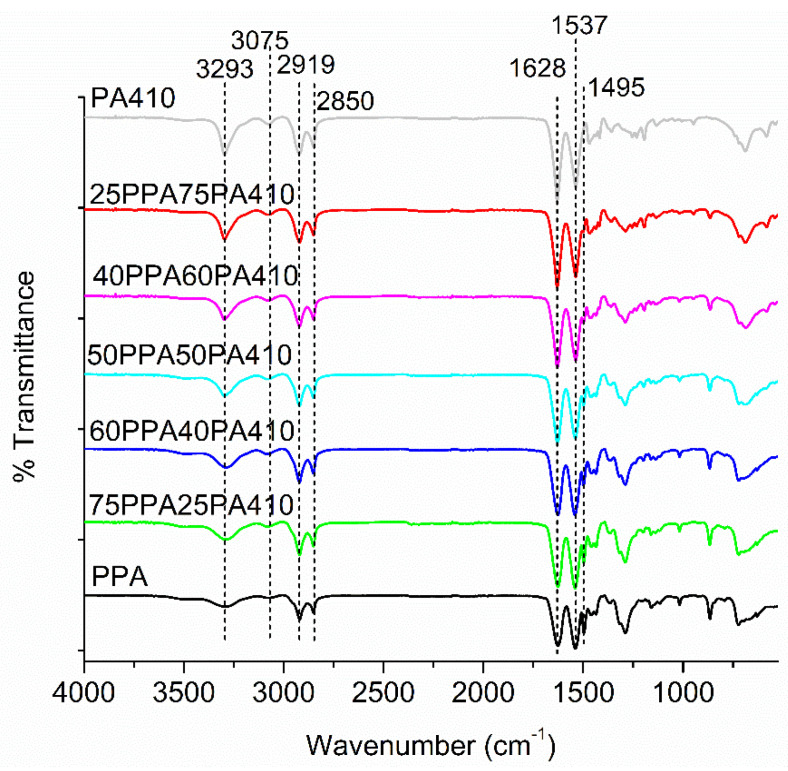
FTIR spectra of neat polymer and the prepared blends.

All the FTIR spectra of the blends can be observed in Figure 10. As reported in previous studies [35,36], both polymers and the blends show similar spectra, due to their nature as polyamides. The peaks at 2850 and 2919 cm^−1^ correspond to vibrations associated with the CH_2_ chains; the peaks at 3293 and 1628 cm^−1^ are associated with amide groups; peaks at 1537, 1495 and 3075 cm^−1^ are associated with benzene rings (which is as the content of PPA decreases, the peak at 1495 cm^−1^ diminishes until it disappears). According to Pagacz et al. [36], a strong peak around 3300 cm^−1^ also indicates a strong hydrogen bonding, which would indicate that hydrogen bonding remains a strong force that promotes the interaction between the two polymers, though it is strongest for neat PA410.

As a novel blend, no rheological properties of them were reported, beyond the measurement of intrinsic viscosity [37,38]. Our research has verified that the addition of PA410 can significantly reduce the viscosity of PPA from the perspective of rheology, and speculates the possible conformational changes of the molecular chain according to the dependence of modulus/viscosity on the composition ratio. The research provides a general base for the processing and modification of this blend system, and indicates that PA410 can expand its uses in terms of applications. 

## 4. Conclusions

Through a simple melt blending modification, blends of PPA and PA410 were found to have superior mechanical properties at a lower processing temperature, compared to neat PPA. The maximum Young’s modulus was reached by the 25PPA/75PA410 blend at 3 GPa; the highest HDT of the blends was that of 25PPA/75PA410 at 136 °C. The changes in glass transition temperature and HDT are also similar to blends of other polyamides with PPA, as well as indicating co-crystallization. The rheological behavior also showed a change of the PPA from a shear thinning fluid to a more Newtonian liquid, thus opening the possibility of a reduced viscosity which could be exploited at industrial settings without an increase in cross polymerization or chain branching. No significant changes in the thermal behavior of the blends were observed when compared to PPA. The results obtained by analyzing these blends, open the possibility of PPA/PA410 blends with an increase in biobased content that maintain good mechanical properties at temperatures above which commodity plastics cannot be employed, increasing the sustainability of PPA, as well as expanding the use of the PA410 in applications where mechanical properties are more demanding

## Figures and Tables

**Table 1:** Coding of produced blends and temperature employed to melt mix them.

Code	wt% of PPA	wt% of PA410	Temperature of Melt Mixing [°C]
PPA	100	0	325
75PPA/25PA410	75	25	305
60PPA/40PA410	60	40	305
50PPA/50PA410	50	50	305
40PPA/60PA410	40	60	305
25PPA/75PA410	25	75	305
PA410	0	100	285

**Table 2:** Enthalpy of fusion and crystallization, percentage of crystallization of the blends, and apparent miscibility or immiscibility.

Samples	Lowest *T_m_* [°C]	Δ*H_m_* [J/m]	*T_c_* [°C]	Δ*H_c_* [J/m]	*χ_c_* [J/m]
PPA	299.2	14.08	259.22	27.5	N/A
75PPA/25PA410	288.56	21.73	248.95	30.78	78.08
60PPA/40PA410	284.61	30.44	243.47	19.32	46.25
50PPA/50PA410	231.34	18.52	191.79	28.01	34.59
40PPA/60PA410	237.51	30.98	201.65	43.25	68.99
25PPA/75PA410	243.1	36.77	209.71	57.6	46.78
PA410	244.21	52.93	220.72	69.07	45.35

**Table 3:** Thermal properties derived from the TGA and HDT analysis of the produced PPA/PA410 blends.

Samples	HDT (0.2% at 0.455 MPa) [°C]	2% Weight Loss Temperature [°C]	5% Weight Loss Temperature [°C]	10% Weight Loss Temperature [°C]	Maximum Degradation Temperature [°C]
PPA	122.30 ±17.97	404.86 ± 8.10	429.71 ± 0.33	441.72 ± 0.01	471.55 ± 1.22
75PPA/25PA410	109.91 ± 11.69	400.91 ± 0.99	417.71 ± 0.55	428.51 ± 0.36	461.47 ± 0.12
60PPA/40PA410	121.89 ± 8.65	398.42 ± 1.08	415.91 ± 0.18	427.01 ± 0.29	459.35 ± 0.49
50PPA/50PA410	121.20 ± 0.12	399.73 ± 1.68	418.29 ± 0.98	429.51 ± 1.17	461.50 ± 1.37
40PPA/60PA410	130.19 ± 2.18	397.58 ± 0.71	416.15 ± 1.70	427.26 ± 1.70	457.46 ± 1.08
25PPA/75PA410	135.99 ± 2.47	398.93 ± 0.66	414.51 ± 0.91	424.85 ± 0.93	455.75 ± 1.01
PA410	160.03 ± 4.58	406.08 ± 0.25	424.17 ± 1.17	433.81 ± 1.68	463.02 ± 4.88

**Table 4:** The glass transition temperature (*T_g_*) obtained from the tan (δ) curves from DMA testing.

Samples	*T_g_* (from DMA) [°C]
PPA	140.77 ± 9.84
75PPA/25PA410	117.00 ± 0.11
60PPA/40PA410	103.16 ± 2.64
50PPA/50PA410	101.39 ± 1.84
40PPA/60PA410	80.63 ± 2.52
25PPA/75PA410	70.32 ± 3.18
PA410	66.44 ± 0.83

## Data Availability

The data is accessible upon request from the corresponding author.

## Supplementary Materials

The following are available online at https://www.mdpi.com/article/10.3390/polym13193391/s1, Figure S1: SEM images of gold-coated impact fracture samples of all PPA/PA410 blends; Figure S2: POM images of all PPA/PA410 blends.

## References

[1] (2019). Plastics—The Facts 2019.

[2] Parker D., Bussink J., van de Grampel H.T., Wheatley G.W., Dorf E.-U., Ostlinning E., Reinking K., Schubert F., Jünger O., Wagener R., Wiley-VCH Verlag GmbH & Co. KGaA (2012). Polymers, High-Temperature. Ullmann’s Encyclopedia of Industrial Chemistry.

[3] Kemmish D. (2011). 3 Semi-Aromatic Polyamides (Polyphthalamides). Practical Guide to High Performance Engineering Plastics.

[4] (2015). D20 Committee Classification System and Basis for Specification for Polyphthalamide (PPA) Injection Molding Materials, D5336-15a.

[5] Hottle T.A., Bilec M.M., Landis A.E. (2017). Biopolymer Production and End of Life Comparisons Using Life Cycle Assessment. Resour. Conserv. Recycl..

[6] Shen L., Worrell E., Patel M.K. (2012). Comparing Life Cycle Energy and GHG Emissions of Bio-Based PET, Recycled PET, PLA, and Man-Made Cellulosics: Modeling and Analysis: Comparing Life-Cycle Energy and GHG Emissions of Bioproducts. Biofuels Bioprod. Biorefin..

[7] Arkema Rilsan^®^ HT. https://www.extremematerials-arkema.com/en/product-families/rilsan-polyamide-11-family/rilsan-ht/.

[8] No Contradiction: VESTAMID^®^ HTplus Both Bio-Based and High-Performing. https://corporate.evonik.com/en/media/press_releases/corporate/no-contradiction-vestamid-htplus-both-bio-based-and-high-performing-103227.html.

[9] Moran C.S., Barthelon A., Pearsall A., Mittal V., Dorgan J.R. (2016). Biorenewable Blends of Polyamide-4,10 and Polyamide-6,10. J. Appl. Polym. Sci..

[10] Leszczyńska A., Kiciliński P., Pielichowski K. (2015). Biocomposites of Polyamide 4.10 and Surface Modified Microfibrillated Cellulose (MFC): Influence of Processing Parameters on Structure and Thermomechanical Properties. Cellulose (Dordrecht, The Netherlands).

[11] Otaegi I., Aranburu N., Iturrondobeitia M., Ibarretxe J., Guerrica-Echevarría G. (2019). The Effect of the Preparation Method and the Dispersion and Aspect Ratio of CNTs on the Mechanical and Electrical Properties of Bio-Based Polyamide-4,10/CNT Nanocomposites. Polymers.

[12] Siciliano A., Severgnini D., Seves A., Pedrelli T., Vicini L. (1996). Thermal and Mechanical Behavior of Polyamide 6/Polyamide 6I/6T Blends. J. Appl. Polym. Sci..

[13] Persyn O., Miri V., Lefebvre J.-M., Ferreiro V., Brink T., Stroeks A. (2006). Mechanical Behavior of Films of Miscible Polyamide 6/Polyamide 6I-6T Blends. J. Polym. Sci. Part B Polym. Phys..

[14] Eersels K.L.L., Groeninckx G. (1996). Influence of Interchange Reactions on the Crystallization and Melting Behaviour of Polyamide Blends as Affected by the Processing Conditions. Polymer.

[15] Cretenoud J., Galland S., Plummer C.J.G., Michaud V., Bayer A., Lamberts N., Hoffmann B., Frauenrath H. (2016). High-Temperature Copolyamides Obtained by the Efficient Transamidation of Crystalline-Crystalline Polyamide Blends. J. Appl. Polym. Sci..

[16] Voigt W. (1889). Ueber die Beziehung zwischen den beiden Elasticitätsconstanten isotroper Körper. Ann. Phys..

[17] Eersels K.L.L., Aerdts A.M., Groeninckx G. (1996). Transamidation in Melt-Mixed Aliphatic and Aromatic Polyamides. 2. Molecular Characterization of PA 46/PA 6I Blends as a Function of the Extrusion Time, Extrusion Temperature, and Blend Composition. Macromolecules (Washington, DC, USA).

[18] Ellis T.S. (1995). Miscibility of Polyamide Blends: Effects of Configuration. Polymer.

[19] Utracki L.A., Wilkie C.A. (2014). Polymer Blends Handbook.

[20] Eersels K.L.L., Groeninckx G., Koch M.H.J., Reynaers H. (1998). Influence of Transreaction Processes on the Morphology of Semicrystalline Aliphatic/Aromatic Polyamide Blends. Polymer.

[21] Grätzl T., van Dijk Y., Schramm N., Kroll L. (2019). Influence of the Automotive Paint Shop on Mechanical Properties of Continuous Fibre-Reinforced Thermoplastics. Compos. Struct..

[22] Shashoua V.E., Eareckson W.M. (1959). Interfacial Polycondensation. V. Polyterephthalamides from Short-Chain Aliphatic, Primary, and Secondary Diamines. J. Polym. Sci. (Hoboken, NJ, USA).

[23] Edgar O.B., Hill R. (1952). The P-Phenylene Linkage in Linear High Polymers: Some Structure–Property Relationships. J. Polym. Sci. (Hoboken, NJ, USA).

[24] Zanjanijam A.R., Hakim S., Azizi H. (2018). Rheological, Mechanical and Thermal Properties of the PA/PVB Blends and Their Nanocomposites: Structure-Property Relationships. Polym. Test..

[25] La Mantia F.P., Morreale M., Botta L., Mistretta M.C., Ceraulo M., Scaffaro R. (2017). Degradation of Polymer Blends: A Brief Review. Polym. Degrad. Stab..

[26] Desio G.P. (1996). Characterization and Properties of Polyphthalamide/Polyamide Blends and Polyphthalamide/Polyamide/Polyolefin Blends. J. Vinyl Addit. Technol..

[27] Yao Z., Sun J., Wang Q., Cao K. (2012). Study on Ester–Amide Exchange Reaction between PBS and PA6IcoT. Ind. Eng. Chem. Res..

[28] Peng W.-M., Tong X., Zhang M.-L., Wang X.-J., Zhang G., Long S.-R., Yang J. (2018). Semiaromatic Polyamide Poly(Hexamethylene Terephthalamide)-*Co*-Polycaprolactam: Thermal and Flame-Retardant Properties. J. Appl. Polym. Sci..

[29] Wang X., Zheng Q., Du L., Yang G. (2008). Influence of Preparation Methods on the Structures and Properties for the Blends between Polyamide 6co6T and Polyamide 6: Melt-Mixing Andin-Situ Blending. J. Polym. Sci. Part B Polym. Phys..

[30] Wang Z., Hu G., Zhang J., Xu J., Shi W. (2017). Non-Isothermal Crystallization Kinetics of Nylon 10T and Nylon 10T/1010 Copolymers: Effect of Sebacic Acid as a Third Comonomer. Chin. J. Chem. Eng..

[31] Kaplan D.S. (1976). Structure–Property Relationships in Copolymers to Composites: Molecular Interpretation of the Glass Transition Phenomenon. J. Appl. Polym. Sci..

[32] Zytner P., Wu F., Misra M., Mohanty A.K. (2020). Toughening of Biodegradable Poly(3-Hydroxybutyrate- *Co* -3-Hydroxyvalerate)/Poly(ε-Caprolactone) Blends by In Situ Reactive Compatibilization. ACS Omega.

[33] Wu F., Misra M., Mohanty A.K. (2019). Super Toughened Poly(Lactic Acid)-Based Ternary Blends via Enhancing Interfacial Compatibility. ACS Omega.

[34] Zanjanijam A.R., Hakim S., Azizi H. (2016). Morphological, Dynamic Mechanical, Rheological and Impact Strength Properties of the PP/PVB Blends: The Effect of Waste PVB as a Toughener. RSC Adv..

[35] Liu Y., Yi J., Cai X. (2011). Effect of a Novel Intumescent Retardant for ABS with Synergist Al(H_2_PO_2_)_3_. Polym. Bull. (Berl. Ger.).

[36] Pagacz J., Raftopoulos K.N., Leszczyńska A., Pielichowski K. (2016). Bio-Polyamides Based on Renewable Raw Materials: Glass Transition and Crystallinity Studies. J. Therm. Anal. Calorim..

[37] Marchildon K. (2011). Polyamides—Still Strong After Seventy Years. Macromol. React. Eng..

[38] Zhang C. (2018). Progress in Semicrystalline Heat-Resistant Polyamides. e-Polymers.

