# Supplementary material for: Evaluating the Performance of a Semiaromatic/Aliphatic Polyamide Blend: The Case for Polyphthalamide (PPA) and Polyamide 4,10 (PA410)
Source: Polymers (Basel). 2021 Oct 2;13(19):3391. doi: 10.3390/polym13193391 (PMC8512554; doi:10.3390/polym13193391)
Supplement: Supplementary file 1 [file polymers-13-03391-s001.zip › polymers-1385627-supplementary.pdf]

polymers

Supplementary Material

Evaluating the Performance of a Semiaromatic/Aliphatic Polyamide Blend: The Case for Polyphthalamide (PPA) and Polyamide 4,10 (PA410)

Mateo Gonzalez de Gortari [1,2], Feng Wu [1], Amar K. Mohanty [1,2] and Manjusri Misra [1,2,*]

[1] School of Engineering, Thornbrough Building, University of Guelph, Guelph, ON N1G 2W1, Canada; mgonza07@uoguelph.ca (M.G.d.G.); fengwu@uoguelph.ca (F.W.); mohanty@uoguelph.ca (A.K.M.)
[2] Bioproducts Discovery and Development Centre, Department of Plant Agriculture, Crop Science Building, University of Guelph, Guelph, ON N1G 2W1, Canada
* Correspondence: mmisra@uoguelph.ca

Citation: de Gortari, M. G.; Wu, F.; Mohanty, A.K.; Misra, M. Evaluating the Performance of a Semiaromatic/Aliphatic Polyamide Blend: The Case for Polyphthalamide (PPA) and Polyamide 4,10 (PA410). *Polymers* **2021**, *13*, 3391. https://doi.org/10.3390/polym13193391

Academic Editor: Silvie Rimpelová

Received: 1 September 2021
Accepted: 28 September 2021
Published: 2 October 2021

Publisher's Note: MDPI stays neutral with regard to jurisdictional claims in published maps and institutional affiliations.

Copyright: © 2021 by the authors. Licensee MDPI, Basel, Switzerland. This article is an open access article distributed under the terms and conditions of the Creative Commons Attribution (CC BY) license (http://creativecommons.org/licenses/by/4.0/).

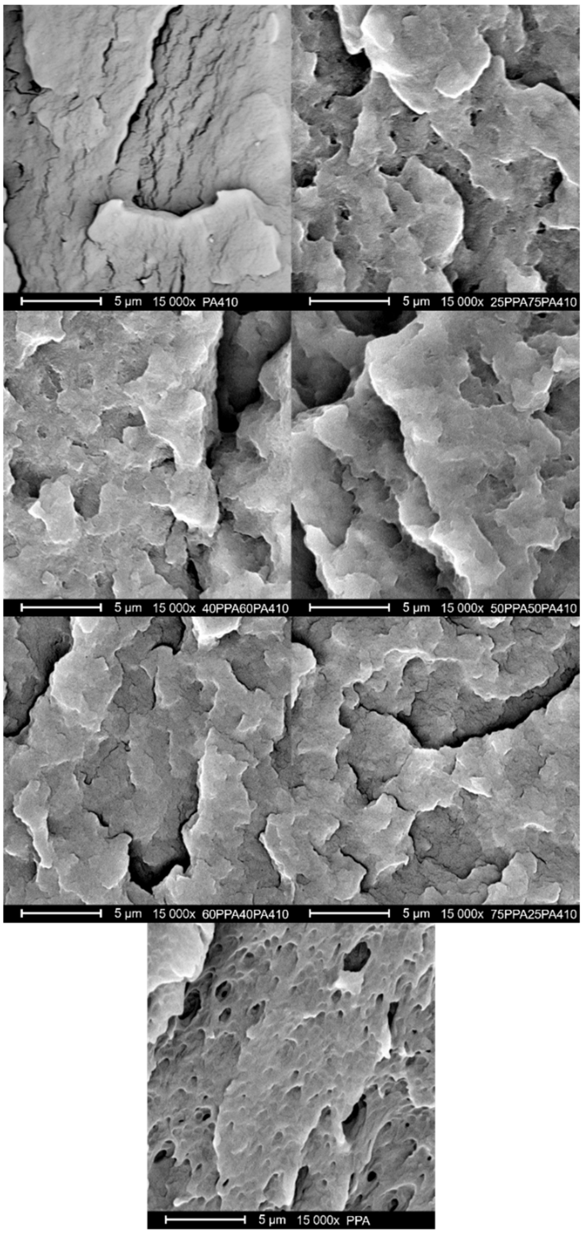

Figure S1. SEM images of gold-coated impact fracture samples of all PPA/PA410 blends.

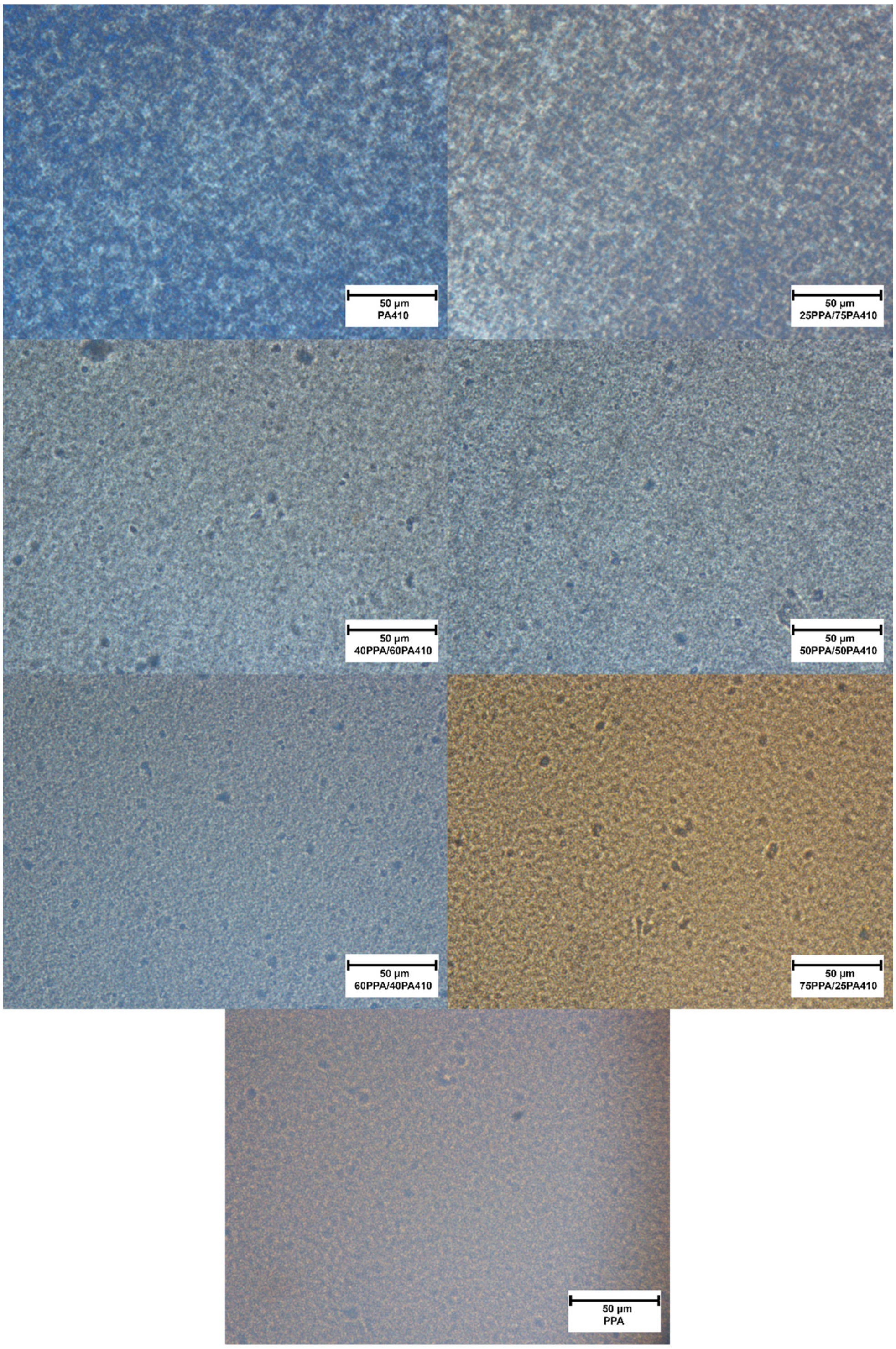

Figure S2. POM images of all PPA/PA410 blends.
